# Exploring the Metabolic and Transcriptomic Profiles of *Tetrastigma hemsleyanum* for Tissue-Specific Compound Accumulation

**DOI:** 10.3389/fpls.2025.1478061

**Published:** 2025-04-02

**Authors:** Lingxia Peng, Hongju Li, Lijun Yang, Zongsuo Liang, Xiaodan Zhang

**Affiliations:** ^1^ Key Laboratory of Plant Secondary Metabolism and Regulation of Zhejiang Province, College of Life Sciences and Medicine, Zhejiang Sci-Tech University, Hangzhou, China; ^2^ Shaoxing Biomedical Research Institute of Zhejiang Sci-Tech University Co., Ltd, Zhejiang Engineering Research Center for the Development Technology of Medicinal and Edible Homologous Health Food, Shaoxing, China

**Keywords:** UPLC-Q-TOF-MSE, RNA-Seq, *Tetrastigma hemsleyanum*, medicinal plant, different parts, accumulation

## Abstract

**Introduction:**

*Tetrastigma hemsleyanum* Diels et Gilg is a medicinal plant known for its diverse pharmacological effects, including anti-inflammatory, anti-tumor, anti-hepatocellular carcinoma, and antipyretic activities. To explore the medicinal components from different parts of the plant and to fully utilize *T. hemsleyanum*, this study investigated the mechanisms underlying the differential accumulation of metabolites in its tuberous roots, fibrous roots, and leaves.

**Methods:**

This study employed a combination of metabolomics and transcriptomics to analyze the metabolic profiles of *T. hemsleyanum*. Using LC-MS/MS technology in positive ion mode, metabolites were identified and quantified in the tuberous roots, fibrous roots, and leaves. Key metabolic pathways were analyzed to understand the spatial distribution of bioactive compounds.

**Results:**

A total of 65 metabolites were identified in the tuberous roots, 203 in the fibrous roots, and 235 in the leaves. The main compounds identified included flavonoids, alkaloids, terpenoids, glycosides, ketones, and amino acids and their derivatives. Flavonoids, glycosides, alkaloids, and terpenoids were strongly accumulated in the tuberous roots, while flavonoid alcohols, glycosides, alkaloids, and terpenoids were predominant in the leaves and fibrous roots. The phenylpropanoid biosynthesis pathway and isoflavonoid biosynthesis were found to play a major role in the pharmacological effects of *T. hemsleyanum*. The glucosinolate pathway and ABC transporters were also identified as key contributors to tissue-specific metabolic accumulation.

**Discussion:**

These results elucidate the molecular mechanisms behind the differential accumulation of metabolites in different parts of *T. hemsleyanum*. The findings provide important insights into the spatial distribution of its bioactive components and their biosynthetic pathways, offering a foundation for further development and utilization of this medicinal plant.

## 1 Introduction

*Tetrastigma hemsleyanum* (also known as the three-leafed green, golden-thread hanging gourd, four-immortals hanging bell, earth arrowroot, and snake’s root, Jiangsu New Medical College, 1977) is a tuberous plant of the Vitaceae family. This species is an endangered medicinal plant endemic to China, primarily distributed in the southern regions, including Zhejiang, Fujian, Jiangxi, Guangdong, Guangxi, Sichuan, Hunan, Yunnan, and Guizhou provinces. It typically grows in humid valleys and forests with high humus content (Wei et al., 2011; Xu et al., 2016). The medicinal value of *T. hemsleyanum* is widely recognized, and it is listed as one of the “Eight Treasures of Zhejiang.” (Hu et al., 2021a, b) However, the differences in the medicinal components across different parts of *T. hemsleyanum* are not well understood, which significantly restricts the development of the *T. hemsleyanum* industry.

The extracts of *T. hemsleyanum* are rich in flavonoids, phenolic acids, glycosides, and alkaloids, and are widely used in clinical treatments for diseases such as high fever, asthma, hepatitis, and lung cancer (Yang et al., 2021). The tuber of *T. hemsleyanum* can be used internally for treating high fever, dysentery, pharyngitis, and hepatitis, and externally for treating snake bites and contusions (Chen et al., 2011; Xiong et al., 2022). Thus, *T. hemsleyanum* is also known as a “plant antibiotic” (Chen, 2018). Domestic and international research on *T. hemsleyanum* mainly focuses on its active medicinal components. Li (Li et al., 2003) identified compounds such as apigenin, quercetin, and naringin-3-O-rutinoside in the tuber of *T. hemsleyanum*. Wu (Wu and Lv, 2006) analyzed the pharmacology, chemical composition, and medicinal properties of *T. hemsleyanum* and found that it has anti-liver cancer, hepatoprotective, and anticancer effects (Xia et al., 2016; Xu et al., 2022). However, research on the systematic composition and distribution of all bioactive components in *T. hemsleyanum* is still limited (Xia et al., 2021).

Untargeted metabolomics is a systematic, unbiased approach used to study the composition and variations of small molecules in biological systems (Chen et al., 2021; Xia et al., 2023; Zhang et al., 2016; Peng et al., 2024b). It has gained widespread application following the development of genomics, proteomics, and transcriptomics. In recent years, untargeted metabolomics, especially those based on high-resolution mass spectrometry techniques, have become powerful tools for analyzing the chemical composition of complex systems, such as traditional Chinese medicine and other natural multi-component systems. This method not only enables comprehensive analysis of metabolites but also, when combined with multivariate statistical methods, can visualize sample differences and quickly screen characteristic differential metabolites. Numerous studies have shown that metabolomics has broad applications in biological sample analysis. For example, Wang (Wang et al., 2017) used metabolomics to identify specific biomarkers in traditional Chinese medicine, Peng (Peng et al., 2024a) identified 88 compounds in the stems and leaves of five species of *Dendrobium* using metabolomics, and a widely targeted metabolite modificomics (WTMM) strategy was developed based on ultra-high-performance liquid chromatography-quadrupole-linear ion trap (UHPLC-Q-Trap) and ultra-high-performance liquid chromatography-quadrupole-Exactive-Orbitrap (UHPLC-QE-Orbitrap), which significantly enhanced the detection sensitivity and identification efficiency of modified metabolites (Yang et al., 2024).

Currently, the tuber of *T. hemsleyanum* is the most widely used medicinal part, while the leaves and other parts of the tuber have not been fully explored for their medicinal components. The distribution patterns and accumulation mechanisms of bioactive components in different parts of *T. hemsleyanum* remain unclear. This study selected tuberous roots, fibrous roots, and leaves of *T. hemsleyanum* from Zhejiang Province and conducted a combined analysis using RNA sequencing (RNA-seq) and high-performance liquid chromatography-tandem mass spectrometry (HPLC-MS/MS). This study provides new insights into the utilization of other parts of *T. hemsleyanum*, as well as the biosynthesis, transport, and regulation of its bioactive metabolites.

## 2 Materials and methods

### 2.1 Plant materials

The *T. hemsleyanum* samples were collected in April 2023 from the *Tetrastigma* cultivation base in Hangzhou, Zhejiang Province. According to the “Zhejiang Province Traditional Chinese Medicine Processing Standards (2015 Edition)” (Zhejiang Food and Drug Administration, 2016), all samples were identified as *T. hemsleyanum* by Associate Professor Xiaodan Zhang from the School of Life Sciences and Pharmaceutical Engineering, Zhejiang Sci-Tech University and subsequently deposited at the Key Laboratory of Plant Secondary Metabolism, Zhejiang Sci-Tech University, China (Specimen Numbers: 20240201001-20240201009).

### 2.2 Experimental sample

The 9 plant samples were divided into 3 groups: the first group of tuberous roots was labeled “TT,” the second group of fibrous roots was labeled “TF,” and the third group of leaves was labeled “TL”. Comparisons were made between the sample groups as TT-TF, TT-TL, and TF-TL, where TT-TF represents the comparison of metabolites between tuberous roots and fibrous roots. Each experimental group consisted of 3 plant samples and 5 biological replicates. Quality control samples (QC) were prepared by mixing equal amounts of the three parts of *T. hemsleyanum*. A QC sample was inserted between every 3 samples to assess the stability and reproducibility of the entire analytical process.

### 2.3 Metabolite extraction

A 100 mg sample powder was placed into an EP tube, and 10 mL of 70% methanol was added. The mixture was precisely weighed and subjected to ultrasonic extraction for 45 minutes. After extraction, the mixture was cooled and then centrifuged at 10,000 rpm for 10 minutes. The supernatant was collected and filtered through a 0.22 µm hydrophobic PTFE filter to obtain the test solution.

### 2.4 UPLC-Q-TOF-MS^E^

Chromatographic conditions: The separation was performed using a Thermo Scientific BEHC18 column (100 mm × 2.1 mm, 1.7 μm). The mobile phase consisted of 0.1% formic acid aqueous solution (A) and 100% acetonitrile (B), with a gradient elution program: 0–1 min, 5% B; 1–3 min, 5%–10% B; 3–6 min, 10%–20% B; 6–16 min, 20%–70% B; 16–20 min, 70%–100% B. The flow rate was set at 0.3 mL/min, and the column temperature was maintained at 30°C. The detection wavelength was set between 200 and 600 nm, and the injection volume of the test solution was 2 μL.

Mass spectrometry conditions: Ionization mode: positive and negative; nebulizer gas: nitrogen; collision gas: argon; full scan range: 50 to 1200 Da; source temperature: 120°C; desolvation temperature: 450°C; cone voltage: 25 V; lock spray standard: 400 ng/mL leucine enkephalin (m/z 554.2615 in negative ion mode) with a flow rate of 5 μL/min. Collision energy: 30 to 70 V.

### 2.5 Total RNA extraction and RNA-Seq

Total RNA was extracted using the Plant Total RNA Extraction Kit (Vazyme). Approximately 80 mg of fresh leaves, fibrous roots, and tuberous roots of *T. hemsleyanum* were quickly ground in liquid nitrogen, then lysed with PSL buffer. RNA was extracted according to the kit’s instructions. The RNA quality was assessed using a NanoDrop One spectrophotometer (NanoDrop Technologies, Wilmington, DE) and a Qubit 3.0 Fluorometer (Life Technologies, Carlsbad, CA, USA). Additionally, RNA was reverse transcribed into cDNA using the Vazyme reverse transcription kit. The extracted *T. hemsleyanum* cDNA samples were sent to Wuhan Benna Co., Ltd. for transcriptome sequencing. Gene functions were annotated using various databases, including NR (NCBI non-redundant protein sequences), KOG (clusters of orthologous protein groups), Pfam (protein families), UniProt (Universal Protein Resource), GO (gene ontology), and KEGG (Kyoto Encyclopedia of Genes and Genomes). Differentially expressed genes (DEGs) were detected using the DESeq function for size factor estimation and the nbinom test. A significance threshold of p < 0.05 and a fold change greater than 2 or less than 0.5 were applied to identify significantly differentially expressed genes.

### 2.7 Quantitative real-time PCR analysis

The primer sequences are provided in the table. These primers were designed based on CDS sequences using Primer 3plus (https://www.primer3plus.com/) and synthesized by Qingke Biotechnology. MDH was used as the reference gene (Liu Y. et al., 2022). Quantitative real-time PCR was performed using specialized reagents (TRAN), and all genes were subjected to three biological replicates in this study (each biological replicate consisting of three technical replicates). Gene expression was calculated using the 2^−ΔΔCt^ method, and specificity of amplification was determined through melting curve analysis.

### 2.8 Statistical analysis

Mass spectrometry data processing was performed using Waters QI software for automatic chromatographic peak identification, peak matching, peak alignment, peak extraction, peak integration, and normalization. The resulting secondary mass spectra were matched with a custom-built database and the online database (MassBank of North America) for metabolite identification and potential compound fragmentation patterns. The data were then input into SMICA and MetaboAnalyst (https://www.metaboanalyst.ca/) for PCA and OPLS-DA analysis, as well as heatmap clustering analysis. Differential metabolites were selected using thresholds of VIP > 1, FC < 0.5/FC > 2, and p-value ≤ 0.05, followed by KEGG pathway analysis. qRT-PCR results were calculated using Excel 365, and the data were expressed as mean ± standard error. One-way analysis of variance (ANOVA) and t-test were performed using IBM SPSS statistical software 26.0 for statistical significance analysis.

## 3 Results

### 3.1 Metabolite quantification analysis

**Figure 1:**
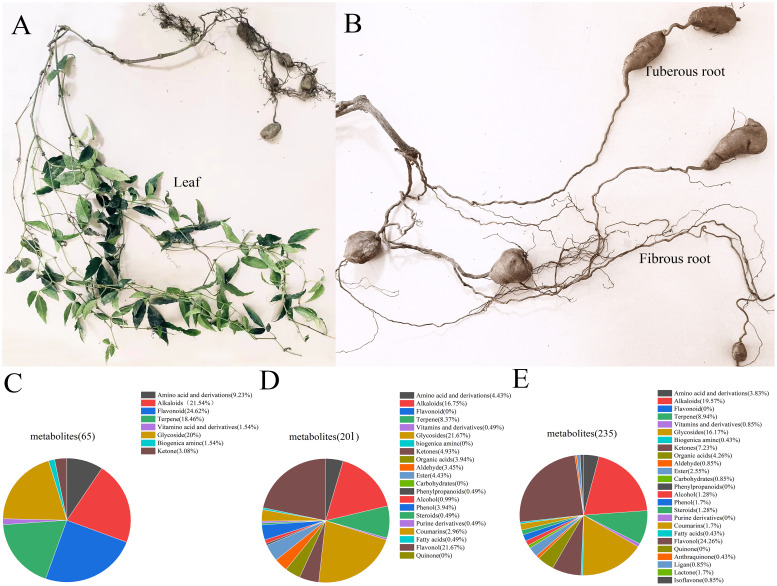
Metabolites identified in different parts of *T. hemsleyanum* in positive ion mode. **(A)** Leaves. **(B)** Tuberous roots and fibrous roots. **(C)** Overall distribution of different metabolite categories in tuberous roots. **(D)** Overall distribution of different metabolite categories in fibrous roots. **(E)** Overall distribution of different metabolite categories in leaves.

The identification results are presented in **Appendix 1** in **Supplementary Material**. In positive ion mode, a total of 65 metabolites were identified in the tuberous roots of *T. hemsleyanum*, 201 metabolites in the fibrous roots, and 235 metabolites in the leaves. **Figures 1A, 1B** show the morphological images of the tuberous roots, fibrous roots, and leaves of *T. hemsleyanum*. The tuberous roots are enriched with chemical components such as flavonoids, alkaloids, glycosides, and terpenes (**Figure 1C**). the fibrous roots and leaves are predominantly rich in flavonoids, glycosides and alkaloids (**Figures 1D, E**).

### 3.2 Principal component analysis and sample correlation analysis of *T. hemsleyanum*

**Figure 2:**
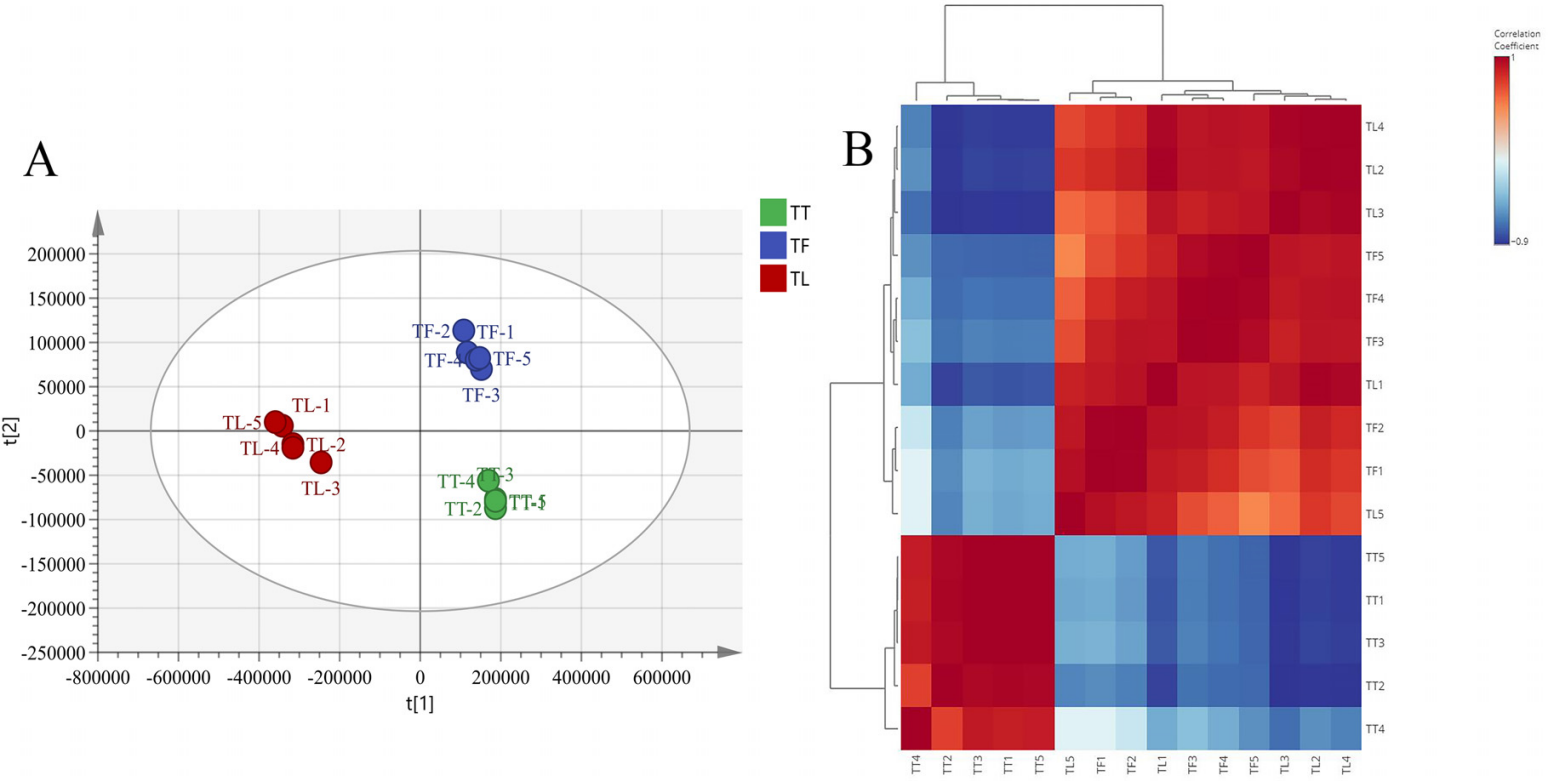
Principal Component Analysis (PCA) and Sample Correlation Analysis of different tissues in *T. hemsleyanum*. PCA analysis of metabolites in different tissue groups based on LC-MS/MS **(A)** and sample-to-sample clustering heat map of different tissues **(B)**. In the LC-MS/MS data analysis, TT, TF, and TL represent the tuberous roots, fibrous roots, and leaves of *T. hemsleyanum*, respectively.

Principal Component Analysis (PCA) analysis reflects the characteristics of metabolomics multidimensional data through several principal components, allowing us to observe differences between groups via the PCA plot. In this study, PCA results showed clear separation among different parts of *T. hemsleyanum*, indicating significant differences in metabolites among the various parts. The results indicate that the samples are separated into three distinct regions without any overlap. In positive ion mode, PC1 = 0.772, PC2 = 0.124, R^2^ = 0.978, Q^2^ = 0.907. This suggests that the samples within each distinct region have specific metabolic profiles, and the metabolites of the samples within each region are relatively similar (**Figure 2A**). The sample correlation heatmap (sample-to-sample clustering) indicated that the metabolite accumulation values among the nine samples of *T. hemsleyanum* exhibited good reproducibility across three biological replicates (**Figure 2B**). Pearson’s correlation coefficient (r) was used as an evaluation index for biological repeatability. When r^2^ approaches 1, it indicates a stronger correlation between the two repeated samples. Additionally, higher correlation coefficients within groups and between different groups suggest more reliable identification of differential metabolites. The results demonstrated that *T. hemsleyanum* exhibited high intragroup correlation, with tuberous roots showing a negative correlation with fibrous roots and leaves, whereas fibrous roots and leaves displayed a strong correlation. These findings confirmed that *T. hemsleyanum* samples from different tissues had good reproducibility, ensuring the reliability of the experimental results.

### 3.3 Differential metabolite screening and compound identification

By performing univariate and multivariate statistical analyses on the metabolites, VIP values from the OPLS-DA model and fold changes (FC) from univariate analysis were obtained. Differential metabolites were selected using a combination of FC, VIP values from the OPLS-DA model, and p-values from t-tests. Thresholds were set at VIP ≥ 1, FC ≥ 2/≤ 0.5, and p-value < 0.05. The selected differential metabolites are listed in **Supplementary Table S2**. In positive ion mode, there were 246 different metabolites between TT and TL, with 87 identified as differential metabolites. Between TT and TF, 211 different metabolites were found, with 86 classified as differential metabolites. For TF vs. TL, 315 different metabolites were detected, with 199 identified as differential metabolites. After screening, 95 differential metabolites were inferred in negative ion mode, as shown in **Supplementary Table S3**. The differential metabolites in positive ion mode are shown in **Appendix 2** in **Supplementary Material**.

### 3.4 Analysis of differential metabolites between tissues

**Figure 3:**
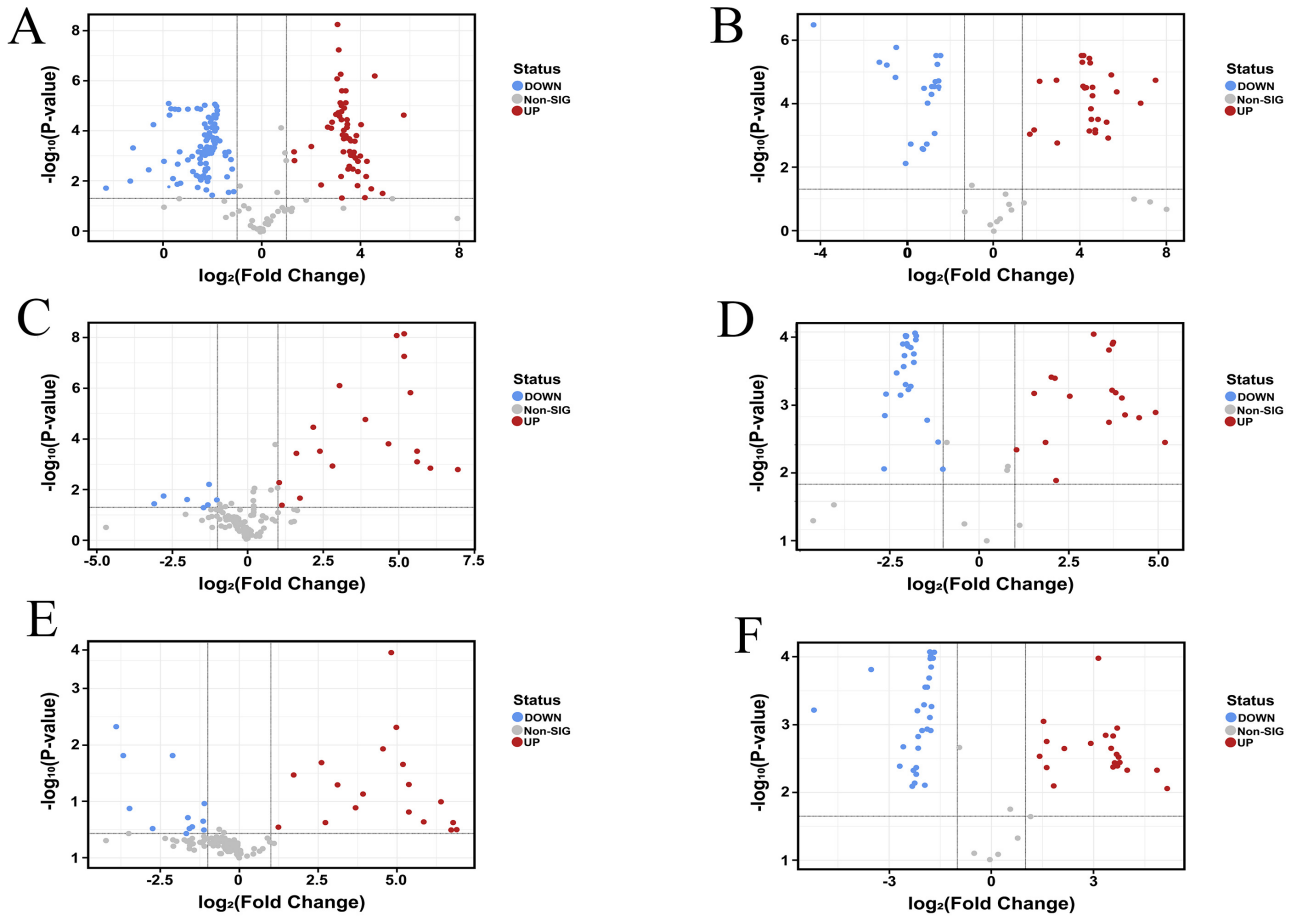
Metabolite Volcano Map. Positive ion mode **(A)** TF vs. TL; **(C)** TT vs. TF; **(E)** TT vs. TL. Negative ion mode **(B)** TF vs. TL; **(D)** TT vs. TF; **(F)** TT vs. TL. Negative ion mode. (“TT” refer to tuberous root. “TF” refer to fibrous root. “TL” refer to leaf.).

The volcano plot visually illustrates the overall distribution of differential metabolites. We analyzed the differences in metabolites across TT, TF, and TL. In the positive ion mode, as shown in **Figure 3A**, compared to TL, TF had a total of 315 metabolites, with 78 significantly upregulated and 127 significantly downregulated. In **Figure 3C**, compared to TF, TT had a total of 211 metabolites, with 14 significantly upregulated and 71 significantly downregulated. As shown in **Figure 3E**, compared to TL, TT had a total of 246 metabolites, with 23 significantly upregulated and 64 significantly downregulated. In the negative ion mode, as shown in **Figure 3B**, compared to TL, TF had 94 metabolites, with 30 significantly upregulated and 29 significantly downregulated. **Figure 3D** shows that compared to TF, TT had 85 metabolites, with 30 significantly upregulated and 17 significantly downregulated. Finally, as shown in **Figure 3F**, compared to TL, TT had 91 metabolites, with 18 significantly upregulated and 34 significantly downregulated.

### 3.5 KEGG enrichment analysis of metabolites

**Figure 4:**
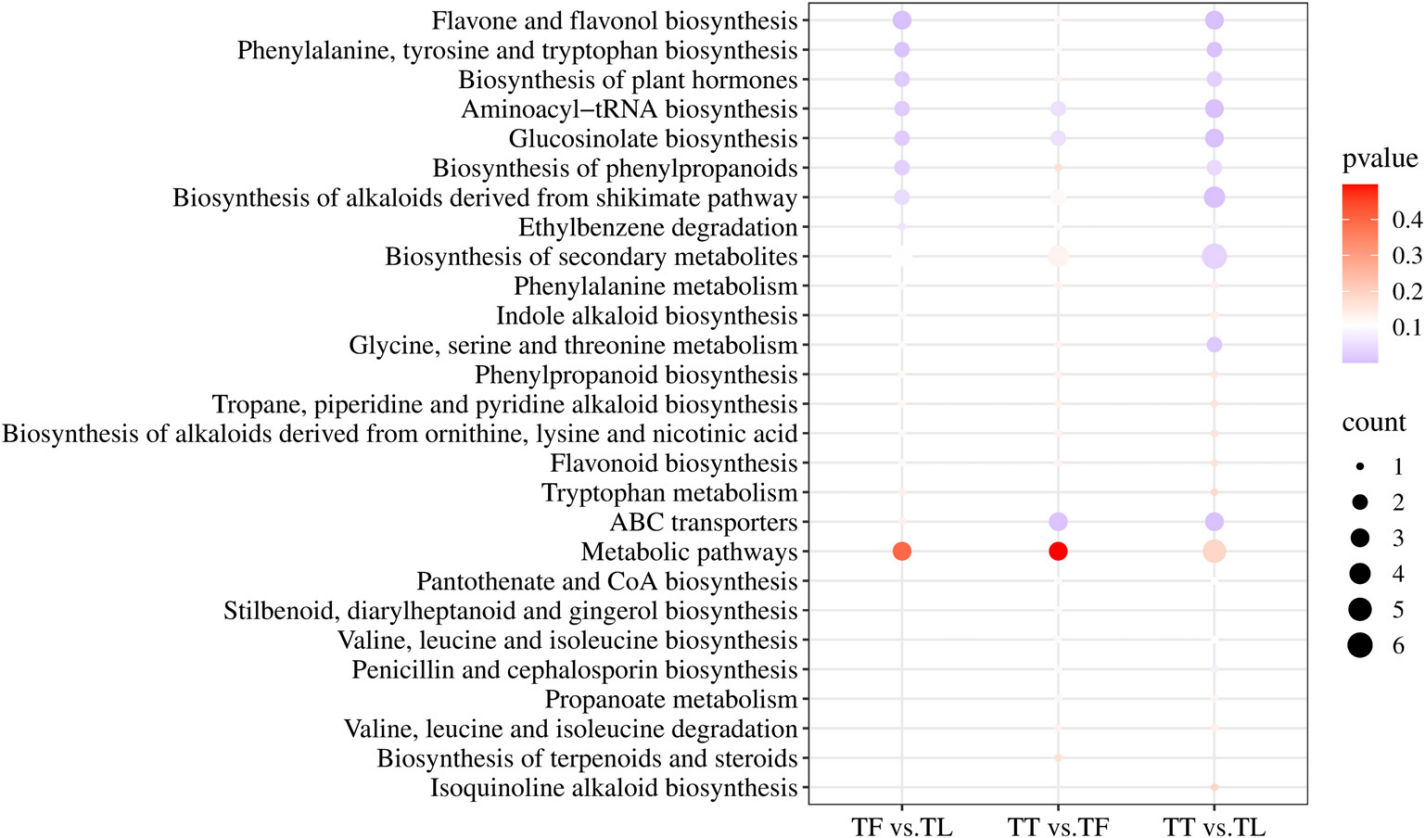
Top 25 KEGG enrichment map of different metabolites.

The KEGG database is a primary resource for studying metabolic pathways, offering potential pathways for the metabolism of carbohydrates, nucleotides, amino acids, and organic matter degradation. It helps understand the interactions between metabolites within the body and the formation of different pathways (Wang et al., 2023; Xiong et al., 2022). This study primarily discusses the enrichment and metabolic pathways of differential metabolites annotated to KEGG pathways among the different comparison groups. The three metabolic sets in positive ion mode, namely TF vs. TL, TT vs. TF, and TT vs. TL, were uploaded to MBRole 2.0 for KEGG id conversion. Among them, 19 out of 36 differential metabolites in the TF vs. TL group obtained corresponding KEGG Ids, 22 out of 39 differential metabolites in the TT vs. TF group obtained corresponding KEGG Ids, and 23 out of 35 differential metabolites in the TT vs. TL group obtained corresponding KEGG Ids. As shown in **Figure 4**, in the TF vs. TL metabolic set, metabolites were significantly enriched in Flavone and flavonol biosynthesis, Phenylalanine, tyrosine and tryptophan biosynthesis, Biosynthesis of plant hormones, Aminoacyl-tRNA biosynthesis, Glucosinolate biosynthesis, Biosynthesis of phenylpropanoids, Biosynthesis of alkaloids derived from shikimate pathway, and Ethylbenzene degradation. In the TT vs. TF metabolic set, metabolites were significantly enriched in ABC transporters, Aminoacyl-tRNA biosynthesis, Glucosinolate biosynthesis, Pantothenate and CoA biosynthesis, Phenylalanine, tyrosine and tryptophan biosynthesis, Stilbenoid, diarylheptanoid and gingerol biosynthesis, Valine, leucine and isoleucine biosynthesis, Biosynthesis of alkaloids derived from shikimate pathway, Penicillin and cephalosporin biosynthesis, and Ethylbenzene degradation. In the TT vs. TL metabolic set, metabolites were significantly enriched in Flavone and flavonol biosynthesis, Biosynthesis of alkaloids derived from shikimate pathway, Aminoacyl-tRNA biosynthesis, Glucosinolate biosynthesis, ABC transporters, Phenylalanine, tyrosine and tryptophan biosynthesis, Glycine, serine and threonine metabolism, Biosynthesis of plant hormones, Biosynthesis of secondary metabolites, Biosynthesis of phenylpropanoids, Penicillin and cephalosporin biosynthesis, and Ethylbenzene degradation. These results indicate that the significantly enriched metabolic pathways of differential metabolites among the tuberous roots, fibrous roots, and leaves of *T. hemsleyanum* may be closely related to its pharmacological effects and properties.

For each group, clustering analysis of all differential metabolites in the significantly enriched pathways can extract useful information to help study the variation patterns of substance content in different groups of potentially important metabolic pathways. As shown in **Table 1**, the fold change (FC) refers to the ratio of metabolites between leaves and rootlets, leaves and rhizomes, and rootlets and rhizomes. The analysis of differential metabolites in the TF vs. TL and TT vs. TL comparison groups shows that the three flavonoids, Apiin, Astragalin, and Rutin, involved in Flavone and flavonol biosynthesis, have higher expression in leaves compared to rootlets and rhizomes. Flavones and flavonols are common flavonoids in the plant kingdom, and the increase in metabolites involved in their biosynthesis pathway ultimately aids in the accumulation of flavonoid compounds. Flavonoids can induce apoptosis in liver cancer, lung cancer, colon cancer SW620, and breast cancer cells. *T. hemsleyanum* flavonoids can inhibit tumor cell proliferation through various pathways, thereby inducing cell apoptosis (Liu Y. et al., 2022). The analysis of differential metabolites in the three comparison groups indicates that the two amino acid compounds, L-Phenylalanine and L-Tryptophan, involved in Phenylalanine, tyrosine, and tryptophan biosynthesis, have significantly higher content in leaves compared to tuberous roots and fibrous roots, and L-Phenylalanine content is significantly higher in tuberous roots than in fibrous roots. The L-Phenylalanine and L-Tryptophan synthesized in this pathway can be used for Glucosinolate biosynthesis. This pathway synthesizes glucosinolates (GS), sulfur-containing glycoside mixtures widely present in the roots, stems, and fruits of Cruciferae plants (Zhou and Yu, 2005). These compounds consist of a β-D-thioglucose residue, a sulfonated oxime group, and an amino acid side chain R group. They can be degraded by myrosinase into nitriles, ascorbigen, and highly physiologically active isothiocyanates (Gu et al., 2012; Groenbaek et al., 2018; Hunzker et al., 2019). Isothiocyanates inhibit cancer cell growth without producing harmful substances to normal cells and are not harmful to the lymphatic system. Glucosinolates can significantly reduce the incidence of stomach, rectal, colon, breast, bladder, prostate cancer, and melanoma (Lin et al., 2015). *T. hemsleyanum*, a rare and precious plant unique to China, can exert broad-spectrum antitumor effects through various pathways. It has been included in the 2015 edition of the “Zhejiang Provincial Standards for Chinese Medicine Processing”. The primary chemical constituents contributing to its anticancer effects may be the metabolites synthesized through the glucosinolate pathway.

**Table 1:** The differential metabolites enriched in the biosynthesis of flavones and flavonols, phenylalanine, tyrosine and tryptophan biosynthesis, and glucosinolate biosynthesis.

Compound name	Comparison of different parts	Formula	FC
Apiin	TF vs.TL	C_26_H_28_O_14_	3.99
	TT vs.TL		5.81E+16
Astragalin	TF vs.TL	C_21_H_20_O_11_	16.13
	TT vs.TL		1.16E+16
Rutin	TF vs.TL	C_27_H_30_O_16_	9.7E+15
	TT vs.TL		9.7E+15
L-Phenylalanine	TF vs.TL	C_9_H_11_NO_2_	12.16
	TT vs.TL		71.29
	TT vs.TF		5.86
L-Tryptophan	TF vs.TL	C_11_H_12_N_2_O_2_	9.75E+15
	TT vs.TL		9.75E+15

TT, TF, and TL represent the tuberous roots, fibrous roots, and leaves of *T. hemsleyanum*, respectively.

### 3.6 Flavonoid profiles in tuberous roots, fibrous roots, and leaves of *T. hemsleyanum*

**Figure 5:**
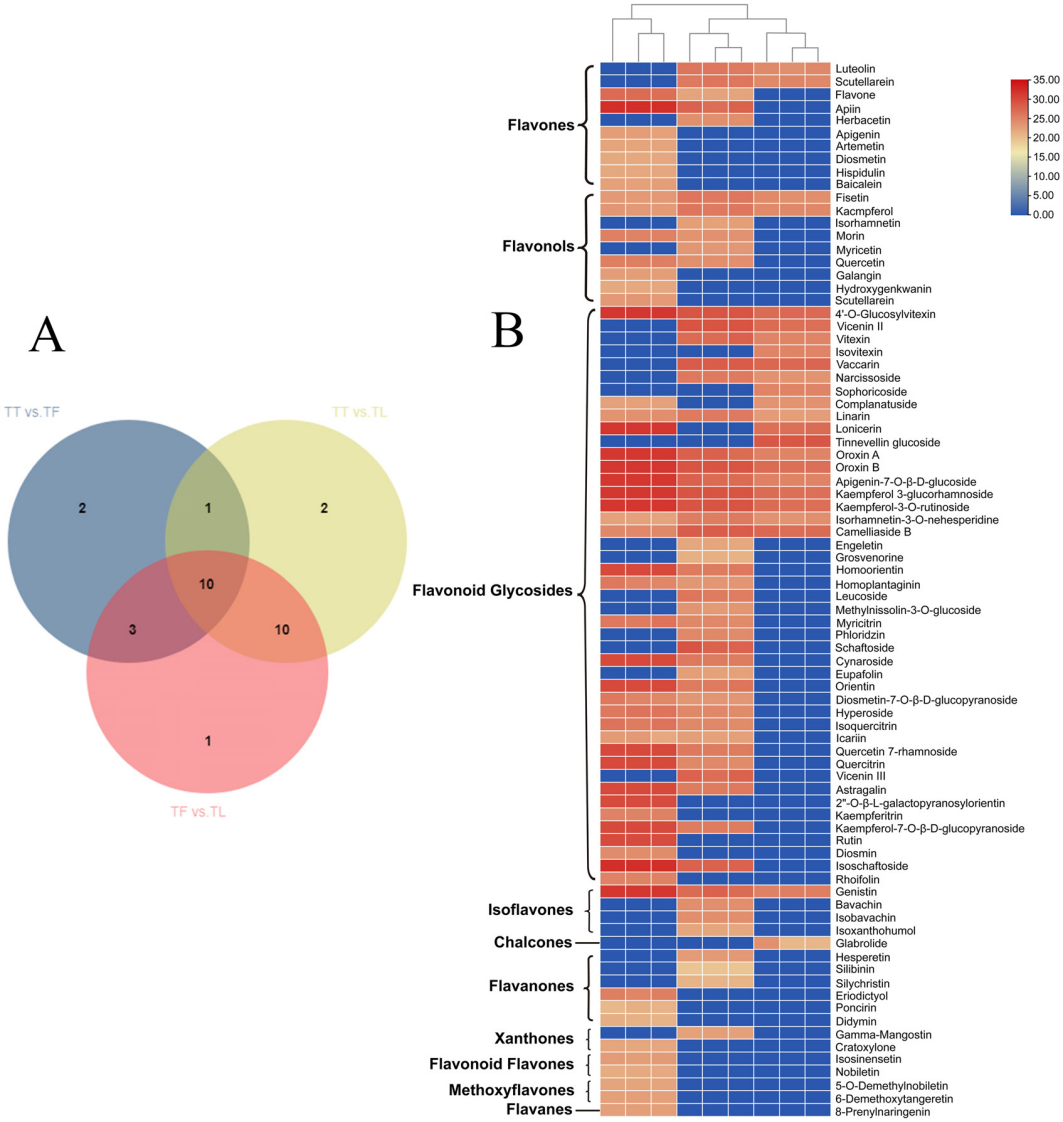
Heatmap visualization of metabolomics data with hierarchical clustering analysis (HCA). **(A)** Venn, and **(B)** Represents the heatmap of flavonoid compounds in different tissue parts of *T. hemsleyanum*. The red color represents the peak value that is relatively large; the blue color represents the peak value that is relatively small; and the gray color represents the metabolite peak value of zero. The upper dendritic structure is clustered according to the degree of metabolite similarity across samples. (“TT” refer to tuberous root. “TF” refer to fibrous root. “TL” refer to leaf.).

We further examined the differential accumulation of flavonoid compounds in various tissue parts of *T. hemsleyanum*. A total of 29 flavonoids with differential accumulation (DAFs) were detected and identified. In the comparison between tuber and fibrous root (TT vs. TF), 16 DAFs were significantly upregulated; in the comparison between tuber and leaf (TT vs. TL), 29 DAFs were significantly upregulated; and in the comparison between fibrous root and leaf (TF vs. TL), 28 DAFs showed significant accumulation. The results indicated that the majority of DAFs were highly accumulated in the leaves of *T. hemsleyanum*. To further investigate the distribution pattern of DAFs, a Venn analysis was performed. The analysis revealed that 29 DAFs were shared across the comparison groups (**Figure 5A**), which included 10 flavones, 9 flavonols, 4 flavonoid glycosides, 4 isoflavones, 1 chalcone, 6 flavanones, 2 Xanthones, 2 flavonoid flavones, 2 methoxyflavones, and 1 flavan. The accumulation patterns of these 10 common DAFs in different tissue parts (**Figure 5B**). These DAFs were predominantly accumulated in the leaf and fibrous root, with some accumulation also observed in the tuber. Further analysis of tissue-specific DAFs revealed that 22 flavonoids accumulated at high concentrations exclusively in the leaves, with negligible levels detected in the tuber and fibrous root. These DAFs included flavones, flavonols, flavonoid glycosides, flavanones, xanthones, flavanolens, methoxyflavones, and flavans. For instance, apigenin, artemetin, diosmetin, hispidulin, and rutin were primarily found in the leaf. In the fibrous root, 18 specific DAFs were detected, including herbacetin, isorhamnetin, methylnissolin-3-O-glucoside, hesperetin, isobavachin, and tinnevellin glucoside. In contrast, only 3 specific DAFs were found in the tuber: isovitexin, sophoricoside, and tinnevellin glucoside. These findings suggest that the distinct distribution and accumulation patterns of DAFs in different tissues may play a key role in the variation of flavonoid content across the tissue parts of *T. hemsleyanum*. To explore the regulatory mechanisms behind these differences, we conducted transcriptome sequencing (RNA-seq) analysis to identify potential pathways or regulatory mechanisms involved.

### 3.7 Phenylalanine, tyrosine and tryptophan biosynthesis, and glucosinolate biosynthesis also showed positive effects on tissue-specific metabolic accumulation

In the KEGG metabolic pathway analysis of *T. hemsleyanum*, the glucosinolate biosynthesis pathway was enriched. Glucosinolates are sulfur-containing secondary metabolites synthesized by plants from one of eight amino acids, including alanine, valine, leucine, isoleucine, phenylalanine, methionine, tyrosine, and tryptophan (International Agency for Research on Cancer Workgroup, 2004). The synthesis of glucosinolates involves the conversion of elongated amino acids to their oxime derivatives, catalyzed by members of the cytochrome P450 (CYP) 79 family (Bak et al., 1999). The oxime is then metabolized to a thiohydroximate, which conjugates with glucuronic acid to form a desulfoglucosinolate, and finally, it undergoes sulfation to yield the glucosinolate (International Agency for Research on Cancer Workgroup, 2004). While glucosinolates themselves do not directly exhibit strong anticancer effects, their hydrolysis products are believed to be primarily responsible for their anticancer activity. This hydrolysis is catalyzed by myrosinase (β-glucosidase, EC 3.2.3.1), which is sequestered in specialized “myrosin cells,” thus physically isolating glucosinolates from their breakdown products in the intact plant (Bones and Rossiter, 1996). In plants, phenylalanine, tyrosine, and tryptophan are central precursors for multiple important biosynthetic pathways, including the synthesis of flavonoids and glucosinolates. Phenylalanine, as a key starting material in the phenylpropanoid pathway, is first deaminated by phenylalanine ammonia-lyase (PAL) to form cinnamic acid, which is then converted into flavonoids, lignins, and other phenolic compounds through a series of enzymatic reactions. Tyrosine plays a supporting role in phenylpropanoid metabolism, participating not only in the synthesis of certain flavonoids and coumarins but also in the formation of other specific phenolic compounds. Tryptophan is a key precursor for the synthesis of indolic glucosinolates. Through a series of modification reactions, including side-chain elongation, oxidation, and glycosylation, tryptophan is converted into biologically active indolic glucosinolates. As an important sulfur-containing secondary metabolite, glucosinolates play a crucial role in plant defense, helping plants resist herbivores and pathogenic microorganisms. Therefore, phenylalanine, tyrosine, and tryptophan are not only fundamental precursors for the synthesis of flavonoids and glucosinolates but also play significant roles in plant physiological functions and ecological adaptation.

### 3.8 Transcriptome sequencing analysis

Transcriptome sequencing was conducted on nine samples from different tissues of *T. hemsleyanum*. The GC content ranged from 44.918% to 46.234%, and more than 95.227% of the bases had a quality score of Q30 or higher (**Table 2**), indicating the high reliability of the RNA-Seq data. Clean reads were assembled, and functional annotations of unigenes were performed by aligning them with databases such as KEGG, NR, UniProt, GO, KOG, and Pfam, yielding 35,874 annotated unigenes. Differential enrichment analysis of these unigenes was carried out across various comparison groups, followed by correlation analysis using metabolomic data obtained through LC-MS/MS.

**Table 2:** Summary of RNA-Seq quality, assembly statistics, and results for 9 sequencing libraries from different *T. hemsleyanum* tissues.

Sample name	Raw reads	Raw bases	Clean reads	Clean bases	Q30 rate	GC content	Mapped reads	Mapped ratio (%)
TF1	43,397,256	6,509,588,400	43,384,734	6,260,287,671	95.429%	45.320%	40,590,608	93.56
TF2	43,382,978	6,507,446,700	43,366,930	6,284,690,429	95.862%	44.918%	39,876,796	91.95
TF3	43,366,026	6,504,903,900	43,349,038	6,222,005,208	96.268%	44.979%	38,799,988	89.50
TL1	43,344,460	6,501,669,000	43,329,424	6,251,681,327	96.305%	45.783%	41,748,968	96.35
TL2	43,384,350	6,507,652,500	43,369,376	6,276,899,695	96.010%	45.822%	41,916,858	96.65
TL3	43,398,852	6,509,827,800	43,385,958	6,287,191,737	96.227%	45.217%	41,884,838	96.54
TT1	43,387,646	6,508,146,900	43,387,642	6,216,396,525	96.788%	46.234%	41,805,762	96.35
TT2	43,388,866	6,508,329,900	43,374,954	6,251,165,105	95.721%	44.971%	38,614,390	89.02
TT3	43,336,600	6,500,490,000	43,327,490	6,172,943,216	95.227%	45.590%	41,281,932	95.28

TT, TF, and TL represent the tuberous roots, fibrous roots, and leaves of *T. hemsleyanum*, respectively.

### 3.9 Differentially expressed genes of tuberous roots, fibrous roots, and leaves of *T. hemsleyanum*

**Figure 6:**
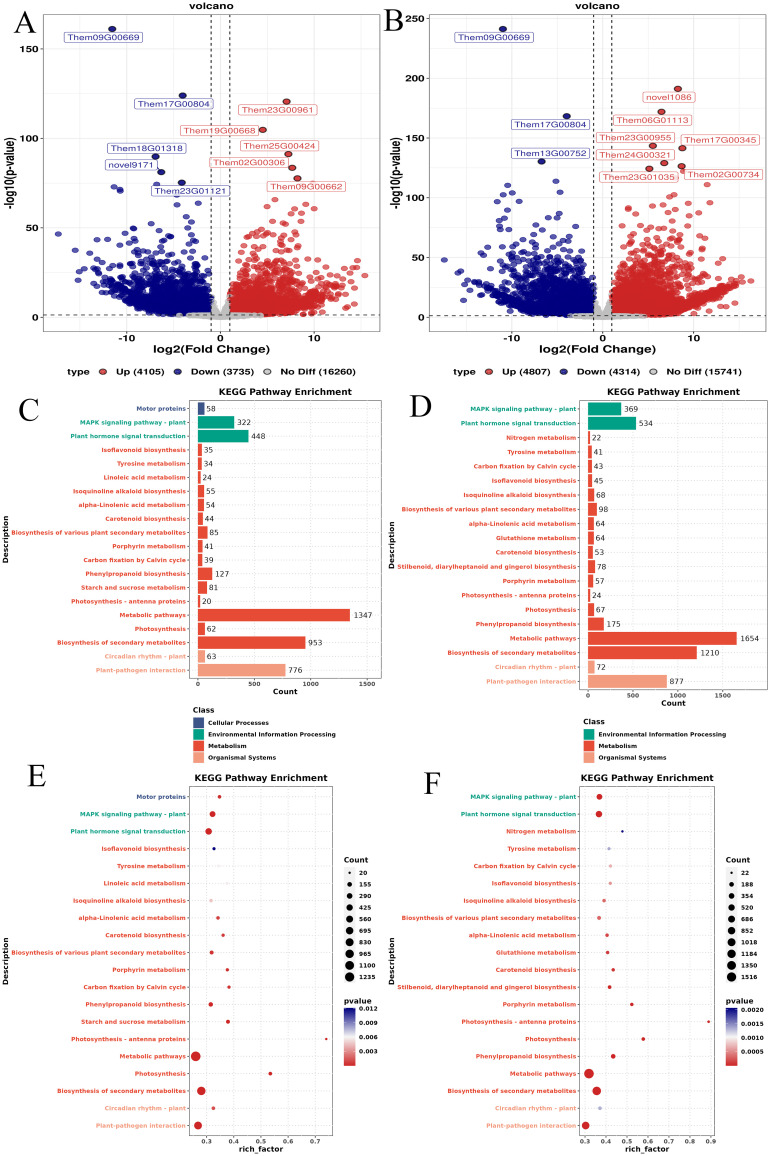
Results of DEGs in different parts of *T. hemsleyanum*. **(A)** Volcano plot of DEGs between leaves and tuberous roots; **(B)** Volcano plot of DEGs between leaves and fibrous roots; **(C)** KEGG classification of DEGs between leaves and tuberous roots; **(D)** KEGG classification of DEGs between leaves and fibrous roots; **(E)** Top 20 enriched KEGG pathways of DEGs between leaves and tuberous roots; **(F)** Top 20 enriched KEGG pathways of DEGs between leaves and fibrous roots. The y-axis represents KEGG pathways, the x-axis represents the enrichment factor, point size indicates the number of DEGs.

A comprehensive analysis of differentially expressed genes (DEGs) in the root tubers, fibrous roots, and leaves of *T. hemsleyanum* revealed results consistent with the differential metabolite analysis. Compared with the leaves, 24,100 DEGs were identified in the tuberous roots, including 3,735 upregulated genes, 4,105 downregulated genes, and 16,260 non-DEGs (**Figure 6A**). Similarly, 24,862 DEGs were identified in the fibrous roots, comprising 4,314 upregulated genes, 4,807 downregulated genes, and 15,741 non-DEGs (**Figure 6B**). KEGG pathway analysis indicated that most DEGs in the tuberous roots (TL vs. TT) were significantly enriched in metabolism-related pathways, including cellular processes, environmental information processing, metabolism, and organismal systems (**Figure 6C**). Likewise, DEGs in the fibrous roots (TL vs. TF) were primarily enriched in pathways related to environmental information processing, metabolism, and organismal systems (**Figure 6D**). The top 20 significantly enriched pathways for DEGs between leaves and tuberous roots are shown in **Figure 6E**. These DEGs were mapped to diverse pathways, such as the mitogen-activated protein kinase (MAPK) signaling pathway, photosynthesis-antenna proteins, plant hormone signal transduction, photosynthesis, metabolic pathways, biosynthesis of secondary metabolites, phenylpropanoid biosynthesis, and plant–pathogen interactions. Among them, the metabolic pathways contained the largest number of DEGs, totaling 1,347, and exhibited the most significant enrichment. In contrast, the isoflavonoid biosynthesis pathway showed the lowest DEG enrichment (**Figure 6E**). Similarly, the top 20 significantly enriched pathways for DEGs between leaves and fibrous roots are presented in **Figure 6F**. These included the MAPK signaling pathway, plant hormone signal transduction, isoflavonoid biosynthesis, photosynthesis-antenna proteins, photosynthesis, phenylpropanoid biosynthesis, metabolic pathways, biosynthesis of secondary metabolites, and plant–pathogen interactions. The metabolic pathways also showed the highest DEG enrichment, totaling 1,654, and exhibited the most significant enrichment level. In contrast, nitrogen metabolism demonstrated the lowest DEG enrichment (**Figure 6F**).

### 3.10 Combined transcriptome and metabolome analysis of *T. hemsleyanum*

**Figure 7:**
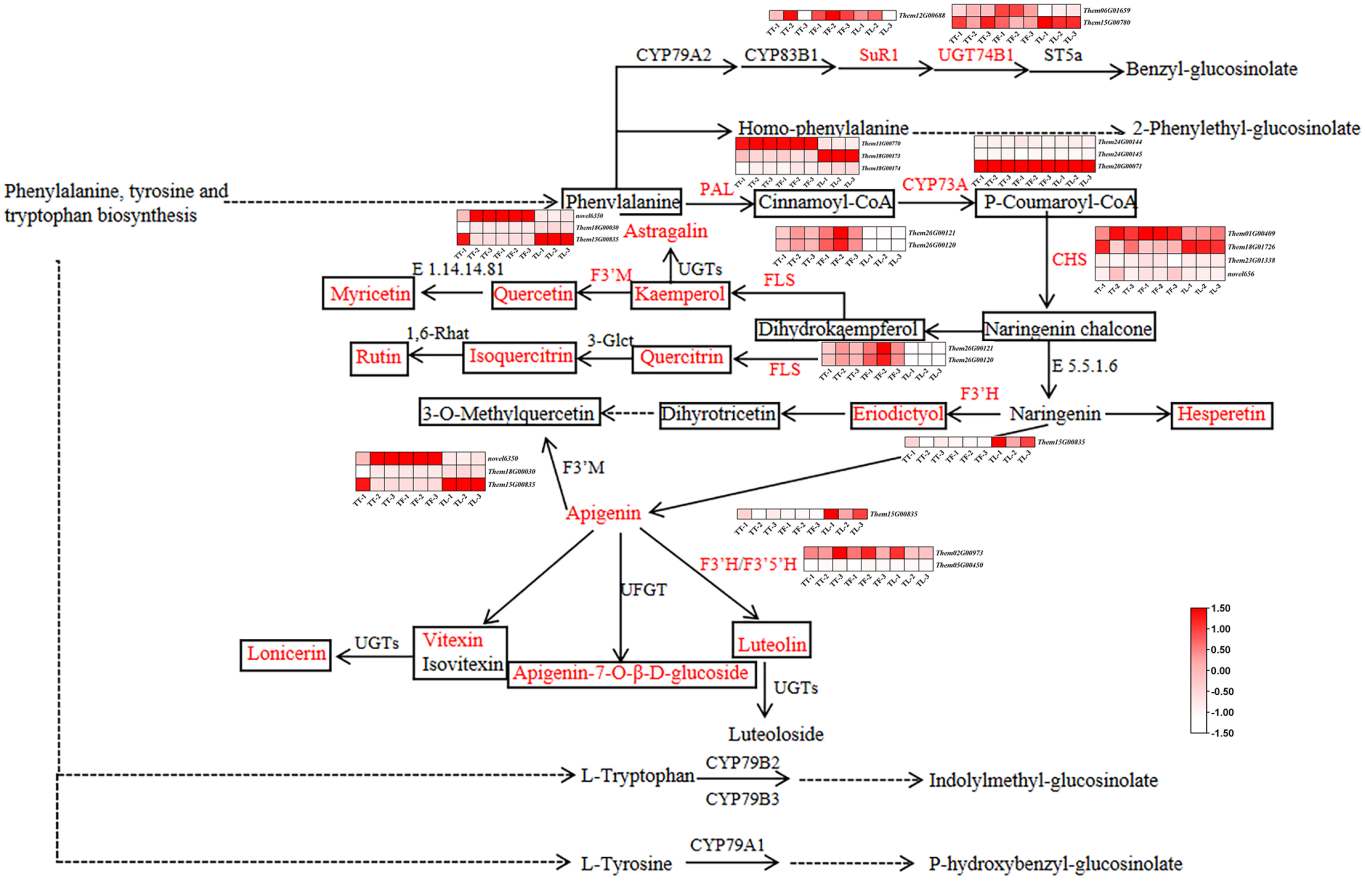
The flavonoid biosynthesis pathway in *T. hemsleyanum*. The heatmap in this pathway shows the expression levels of differentially expressed genes (DEGs). The boxes represent metabolites, with red indicating flavonoids that are significantly accumulated in *T. hemsleyanum*. In the heatmap, red indicates genes that are significantly upregulated, and white indicates genes that are significantly downregulated. In each figure, TT, TF, and TL represent the tuberous roots, fibrous roots, and leaves of *T. hemsleyanum*, respectively. The metabolites and enzymes highlighted in red in the figure are the ones we detected and identified in our experiment.

**Figure 8:**
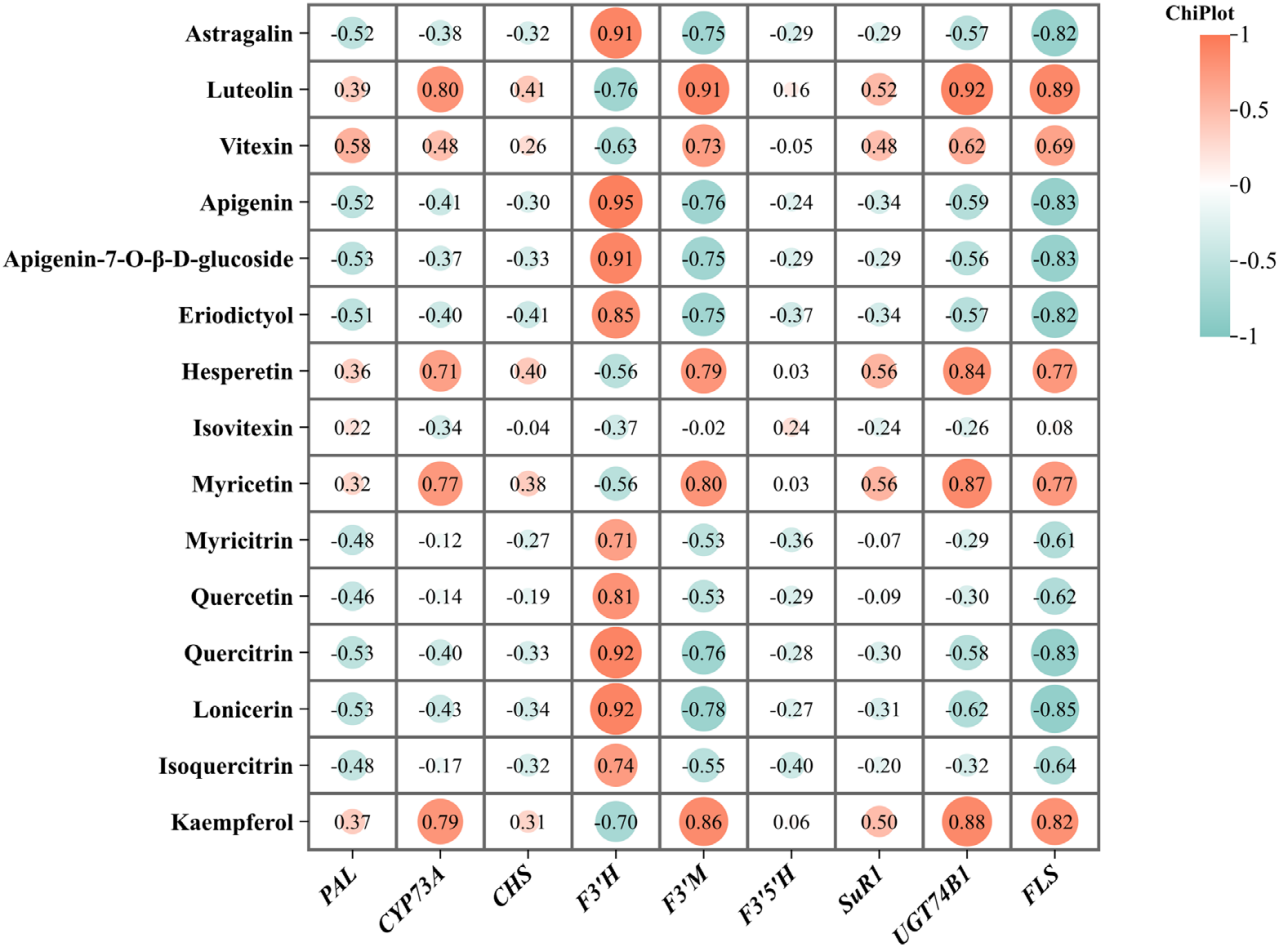
The relationship between flavonoid monomers and the expression of genes related to their biosynthesis.

A combined analysis of RNA-Seq and LC-MS/MS data was conducted through Pearson correlation analysis. Differentially expressed genes (DEGs) and related metabolites involved in the flavonoid biosynthesis pathway of *T. hemsleyanum* were identified. Since the flavonoid biosynthesis, phenylalanine, tyrosine and tryptophan biosynthesis, and glucosinolate biosynthesis pathways share similar metabolites and all contribute to the medicinal effects of *T. hemsleyanum*, we illustrated these three pathways in **Figure 7**. We further analyzed the genes and metabolites significantly enriched in these pathways. As shown in **Figure 7**, two genes encoded by FLS were significantly upregulated in the tuberous and fibrous roots compared to the leaves. Among the three PAL genes, *Them11G00770* was significantly upregulated in the tuberous and fibrous roots compared to the leaves, while Them18G00173 was significantly upregulated in the leaves. CYP73A, encoded by *Them20G00071*, significantly regulates the production of flavonoid compounds in various parts of *T. hemsleyanum*. In JHK, PAL converts phenylalanine to cinnamoyl-CoA, which is then catalyzed by CYP73A to form p-coumaroyl-CoA. Of the four CHS genes, *Them01G00409* was significantly upregulated in the tuberous and fibrous roots compared to the leaves, while *Them18G01726* was significantly upregulated in the leaves. The genes encoding F3’H were significantly upregulated in the leaves compared to the tuberous and fibrous roots. Among the three genes encoding F3’M, *novel6350* was significantly upregulated in the tuberous and fibrous roots, while *Them15G00835* was significantly upregulated in the leaves. Next, Pearson correlation analysis was performed between the metabolite intensities of flavonoid monomers and the typical FPKM values of key genes involved in flavonoid biosynthesis. The results showed a high correlation between the expression of F3’H and eight flavonoids (Astragalin, Apigenin, Apigenin-7-O-β-D-glucoside, Eriodictyol, Quercetin, Quercitrin, and Lonicerin) (**Figure 8**). Furthermore, three flavonoids (Luteolin, Myricetin, and Kaempferol) were highly correlated with the expression of F3’M. Four flavonoids (Luteolin, Hesperetin, Myricetin, and Kaempferol) showed a strong correlation with UGT74B1 gene expression. Two flavonoids (Luteolin and Kaempferol) exhibited a high correlation with FLS gene expression.

### 3.11 Quantitative real-time PCR verification

**Figure 9:**
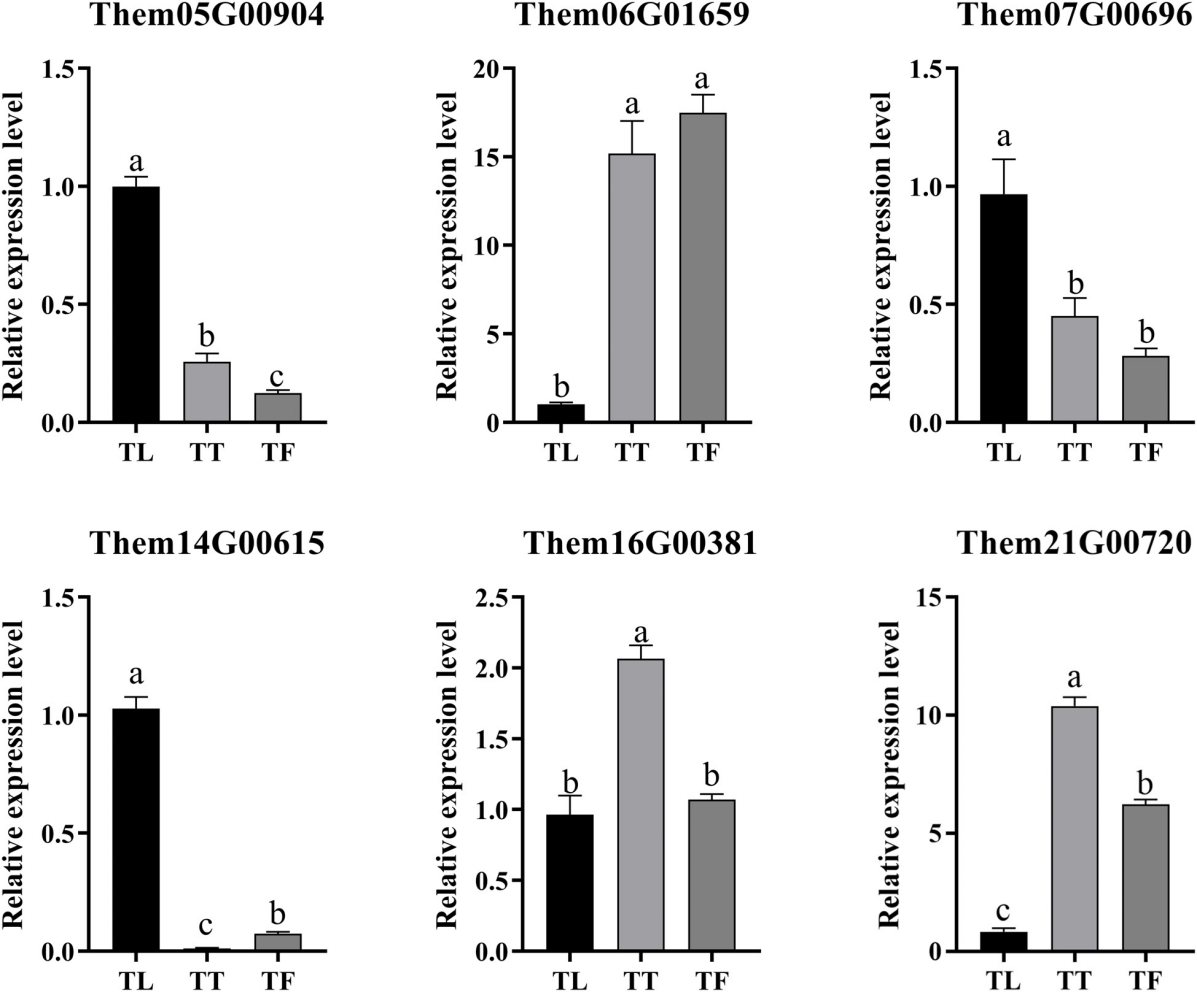
The relative expression levels of six differentially expressed genes (DEGs) involved in flavonoid biosynthesis in the tuberous roots, fibrous roots, and leaves of *Tetrastigma hemsleyanum*. Data are presented as the mean ± standard deviation (SD) from three biological replicates. Different letters (a, b, c) indicate significant differences between groups (P < 0.05), while the same letter indicates no significant difference between groups. TT, TF, and TL represent the tuberous roots, fibrous roots, and leaves of *T. hemsleyanum*, respectively.

To assess the reliability of the transcriptome data in gene expression analysis, we conducted qRT-PCR validation of six key genes involved in flavonoid biosynthesis identified in this study, based on the RNA-Seq results. These genes primarily include differentially expressed genes. The qRT-PCR results showed a consistent trend in gene expression with the RNA-Seq data (**Figure 9**). The findings demonstrate the high reliability of the transcriptome sequencing data (**Table 3**). These findings demonstrate the high reliability of the transcriptome sequencing data (**Table 3**). Primer sequences of DEGs in different parts of *T. hemsleyanum*.

**Table 3:** Primer sequences of DEGs in different parts of *T. hemsleyanum*.

Gene ID	Primer F (5’-3’)	Primer R (5’-3’)
MDH	TGTTGCTACGACTGATGT	CCTGAGACTTGTAGATGGAA
Them06G01659	GTGGAGGAGATTTGGGAAG	CATTCCTCCTGATCTCTTCG
Them16G00381	GTACTGGAGATGTTGTGACC	CGGTCTTCCTAGCATAAGC
Them21G00720	ATCCTGAGGCTACTCTAAGG	GTGTACACCTTCATACCACC
Them10G00819	GAGGACACAAGATCACCTTC	GATATCGGAGGCAGTTTCAG
Them07G00696	GTGATGGACGAAGAGAGTG	GAGGATCCAGAAGTAGTCCT
Them17G01024	CCCAAGAGTGATGAAGAAGG	AGAGGAAAAGGAGGGTGTAG
Them14G00615	CCTTTCTCTGCTAAGACTCC	GTCGGATTCATCTATTCCGG

## 4 Discussion

Currently, in the production and processing of Chinese medicinal herbs, most medicinal materials are used from a single part of the plant, while non-medicinal parts are often discarded. *T. hemsleyanum* has high medicinal value, and all parts of the plant are usable. However, there are no literature reports on the effective components and their differences in the fibrous roots and leaves, which greatly restricts the development of *T. hemsleyanum* (Bian, 2022).

*T. hemsleyanum*, a medicinal plant native to the southern regions of China, was historically used to treat diseases such as pneumonia, asthma, hepatitis, rheumatic pain, trauma injuries, eczema, and snake bites (National Administration of Traditional Chinese Medicine, 1999). Modern pharmacological studies have demonstrated that *T. hemsleyanum* possesses significant medicinal value, with pharmacological effects including, but not limited to, anti-tumor (Liu Y. et al., 2022), anti-viral and anti-inflammatory (Ju et al., 2021), antipyretic (Guo et al., 2018), analgesic (Ye et al., 2020), and immune-regulatory (Lu et al., 2022) activities. Liver disease is common and prevalent in China, and Western medicine often has limited effectiveness. At this time, *T. hemsleyanum* plays a role in liver protection and spleen nourishment (Zhou, 2013). During the outbreak of the novel coronavirus pneumonia at the end of 2019, He Qin and others achieved certain results using a combination of traditional Chinese and Western medicine, where *T. hemsleyanum* was used as an important component in the Chinese medicinal formula for treating COVID-19, playing a significant role in human health (He et al., 2020).

This study utilized metabolomics technology to analyze the metabolome of different parts of *T. hemsleyanum* from the Hangzhou region. The metabolite analysis results indicate that *T. hemsleyanum* contains flavonoids, amino acids and their derivatives, phenolic acids, alkaloids, organic acids, glycosides, sugars, and terpenoids in its various parts. Among these, flavonoids are the most abundant and active, serving as common pharmacologically active components and quality control indicators for *T. hemsleyanum* (Xu et al., 2022; Lan et al., 2022). Flavonoids exhibit broad activities, including anti-tumor, anti-inflammatory, and neuroprotective effects (Li et al., 2021). The roots, stems, and leaves of *T. hemsleyanum* all contain rich amounts of flavonoid active components, which are currently one of the most thoroughly studied classes of compounds in *T. hemsleyanum* (Hu et al., 2018). Modern pharmacological research confirms that the effective anti-tumor components in *T. hemsleyanum* are predominantly flavonoids (Zhong et al., 2019; Wu et al., 2018; Ding and Ji, 2011). Bian Zhihui (Bian, 2022) established an HPLC method for the quantification of four components—chlorogenic acid, isoquercitrin, hesperidin-3-O-rhamnoside, and quercetin—as quality control markers for *T. hemsleyanum*. Chlorogenic acid and quercetin are found in higher concentrations in the leaves than in the roots, while isoquercitrin levels are similar in both parts. Jiang Mengdan (Jiang et al., 2020) and others found that in the underground parts of *T. hemsleyanum*, indicators such as resveratrol, piceatannol, and stilbene glycosides are most concentrated in the fibrous roots and can potentially replace the roots in health products and drug development.

To systematically investigate the distribution and accumulation patterns of flavonoids in different parts of *T. hemsleyanum*, as well as the biosynthesis pathways of phenylalanine, tyrosine, tryptophan, and glucosinolates, we integrated metabolomics (LC-MS/MS) and transcriptomics to explore the biosynthesis mechanisms of flavonoids in *T. hemsleyanum*. Our findings reveal significant interconnections between the flavonoid biosynthesis pathway and those of phenylalanine, tyrosine, tryptophan, and glucosinolates, highlighting the complex regulatory relationships within plant metabolic networks. Phenylalanine serves as a precursor for several important metabolites in plants, including flavonoids and glucosinolates. In flavonoid biosynthesis, phenylalanine is first converted to cinnamic acid by phenylalanine ammonia-lyase (PAL), which is then further transformed into various flavonoid compounds, such as quercetin and kaempferol, through subsequent enzymatic reactions. In the biosynthesis of glucosinolates, phenylalanine is converted to thiol-containing amino acids via the P450-dependent pathway, ultimately leading to the formation of glucosinolates. Notably, the biosynthesis of flavonoids and glucosinolates intersects at several enzymatic steps. For example, key P450 enzymes, such as CYP73A (cinnamate-4-hydroxylase) and CYP79B2 (phenylalanine aminotransferase), are involved in both pathways, suggesting cross-regulation in the conversion of phenylalanine-derived metabolites. Moreover, many genes and metabolites involved in flavonoid and glucosinolate biosynthesis share common regulatory factors, such as plant hormones and environmental stress responses. These factors regulate the expression of specific enzymes, further influencing the accumulation of flavonoids and glucosinolates. Certain environmental conditions, for instance, can simultaneously promote the synthesis of both flavonoids and glucosinolates, enhancing the plant’s ability to resist stress and diseases.

In conclusion, our research demonstrates that different parts of *T. hemsleyanum* hold significant medicinal potential. By combining metabolomics and multivariate analysis, we have differentiated the metabolic characteristics of various plant parts and, in conjunction with transcriptomic data, enhanced our understanding of their medicinal value at both the metabolite and gene levels. However, *T. hemsleyanum* is primarily sourced from wild populations, which are struggling to meet growing demand due to slow natural growth and overharvesting, leading to the species’ near extinction (Peng et al., 2016). Despite its broad range of uses and considerable medicinal value, *T. hemsleyanum* is not yet included in the 2020 edition of the Pharmacopoeia of the People’s Republic of China. Through continued research, we aim to improve cultivation methods and develop advanced processing and purification techniques, unlocking further potential for *T. hemsleyanum* in the future.

## 5 Conclusions

This study employed UPLC-Q-TOF-MS^E^ technology for the qualitative analysis of differential metabolites in different parts of *T. hemsleyanum*. Additionally, differential expression analysis of secondary metabolites provided a clearer understanding of the similarities and differences in secondary metabolites among the three parts. It was found that the highest number of flavonoids were identified in the leaves. The main types of differential metabolites identified in this experiment were flavonoids, amino acids and their derivatives, phenolic acids, and alkaloids. KEGG enrichment analysis of the differential metabolites revealed key metabolic pathways, including Flavone and flavonol biosynthesis, Phenylalanine, tyrosine, and tryptophan biosynthesis, and Glucosinolate biosynthesis. Furthermore, the combination of transcriptome data was used to explore the mechanisms of flavonoid accumulation in the tuberous roots, fibrous roots, and leaves of *T. hemsleyanum* and their associated flavonoid metabolic pathways. The study also mapped the biosynthetic pathways of flavonoids, phenylalanine, tyrosine, and tryptophan biosynthesis, and glucosinolate biosynthesis. This research provides valuable insights into the medicinal efficacy of *T. hemsleyanum* and offers a new theoretical basis for the medicinal use of the whole plant.

## Data Availability

The original contributions presented in the study are included in the article/**Supplementary Material**. Further inquiries can be directed to the corresponding author/s.
