# Supplementary material for: Exploring the Metabolic and Transcriptomic Profiles of Tetrastigma hemsleyanum for Tissue-Specific Compound Accumulation
Source: Front Plant Sci. 2025 Apr 2;16:1478061. doi: 10.3389/fpls.2025.1478061 (PMC12000079; doi:10.3389/fpls.2025.1478061)
Supplement: Supplementary file 3 [file Table3.docx]

**Supporting Information**

**Article title:** Exploring the Metabolic and Transcriptomic Profiles of *Tetrastigma hemsleyanum* for Tissue-Specific Compound Accumulation

**Authors:** Lingxia Peng^1^, Hongju Li^1^, Lijun Yang^1^, Zongsuo Liang^1,2^, Xiaodan Zhang^1*^

The following Supporting Information is available for this article:

Table S1. OPLS-DA validation of samples from different parts in positive ion mode

Table S2. Statistics of difference metabolites analysis results

Table S3. A total of 96 different metabolites identified in negative ion mode.

Table S1. OPLS-DA validation of samples from different parts in positive ion mode

| Group | Ion mode | R²Y | Q² |
| --- | --- | --- | --- |
| TT vs TL | positive | 0.998 | 0.945 |
|  | negative | 0.995 | 0.99 |
| TT vs TF | positive | 0.993 | 0.883 |
|  | negative | 0.998 | 0.994 |
| TL vs TF | positive | 0.999 | 0.99 |
|  | negative | 0.999 | 0.994 |

Table S2. Statistics of difference metabolites analysis results

| Compared Groups | Num. of Total Ident | Num. of Total Sig | Num. of Sig Up | Num. of Sig down |
| --- | --- | --- | --- | --- |
| TT vs.TL_neg | 91 | 52 | 18 | 34 |
| TT vs.TL_pos | 246 | 87 | 23 | 64 |
| TT vs.TF neg | 85 | 47 | 30 | 17 |
| TT vs.TF pos | 211 | 86 | 14 | 71 |
| TF vs.TL neg | 94 | 59 | 30 | 29 |
| TF vs.TL pos | 315 | 199 | 78 | 121 |

Table S3 A total of 96 different metabolites identified in negative ion mode.

|  | Tentative identification | Adducts | T_R_(min) | Formula | Exp.(m/z) | Mass error  (ppm) | Different parts | | |
| --- | --- | --- | --- | --- | --- | --- | --- | --- | --- |
|  |  |  |  |  |  |  | TT | TF | TL |
| 1 | Epicatechin | M-H | 4.77 | C15H14O6 | 289.07 | -1.31 |  | ✔ | ✔ |
| 2 | Epigallocatechin | M-H | 1.9 | C15H14O7 | 305.07 | -0.6 |  | ✔ |  |
| 3 | Procyanidin B1 | M-H | 4.01 | C30H26O12 | 577.14 | -0.45 |  | ✔ |  |
| 4 | Procyanidin B2 | M-H | 4.01 | C30H26O12 | 577.14 | -0.45 |  | ✔ |  |
| 5 | Rutin | M-H | 5.47 | C27H30O16 | 609.15 | 0.01 |  | ✔ |  |
| 6 | Taxifolin | M-H | 1.9 | C15H12O7 | 303.05 | 0.59 |  | ✔ |  |
| 7 | Astragalin | M-H | 8.65 | C21H20O11 | 447.09 | 0.02 | ✔ |  |  |
| 8 | Homoorientin | M-H | 8.65 | C21H20O11 | 447.09 | 0.02 | ✔ |  |  |
| 9 | Kaempferol-7-O-beta-D-glucopyranoside | M-H | 8.65 | C21H20O11 | 447.09 | 0.02 | ✔ |  |  |
| 10 | Orientin | M-H | 8.65 | C21H20O11 | 447.09 | 0.02 | ✔ |  |  |
| 11 | Quercetin 7-rhamnoside | M-H | 8.65 | C21H20O11 | 447.09 | 0.02 | ✔ |  |  |
| 12 | Quercitrin | M-H | 8.65 | C21H20O11 | 447.09 | 0.02 | ✔ |  |  |
| 13 | Naringenin chalcone | M-H | 11.25 | C15H12O5 | 271.06 | -0.05 |  | ✔ | ✔ |
| 14 | Naringenin | M-H | 11.25 | C15H12O5 | 271.06 | -0.05 |  | ✔ | ✔ |
| 15 | (+)-Catechin Hydrate | M-H | 4.77 | C15H14O6H2O | 289.07 | -1.31 |  | ✔ | ✔ |
| 16 | (-)-Gallocatechin | M-H | 1.9 | C15H14O7 | 305.07 | -0.6 |  | ✔ | ✔ |
| 17 | Isobutyrylshikonin | M-CH2+OH | 9.19 | C20H22O6 | 361.13 | -1.54 |  | ✔ |  |
| 18 | Pogostone | M-H | 1.22 | C12H16O4 | 223.1 | -0.21 |  | ✔ |  |
| 19 | Paeonolide | M-H | 7.98 | C20H28O12 | 459.15 | -0.89 |  | ✔ |  |
| 20 | Danshensu | M-H | 1.05 | C9H10O5 | 197.05 | -0.63 |  | ✔ | ✔ |
| 21 | Ethyl gallate | M-H | 1.05 | C9H10O5 | 197.05 | -0.63 |  | ✔ | ✔ |
| 22 | 5-Acetylsalicylic acid | M-H | 1.05 | C9H8O4 | 179.03 | -0.89 |  | ✔ | ✔ |
| 23 | Caffeic acid | M-H | 1.05 | C9H8O4 | 179.03 | -0.89 |  | ✔ | ✔ |
| 24 | Diffractic Acid | M-H | 1.86 | C20H22O7 | 373.13 | -1.08 |  | ✔ | ✔ |
| 25 | Cianidanol | M-H | 4.77 | C15H14O6 | 289.07 | -1.31 |  | ✔ | ✔ |
| 26 | Ginkgolide K | M-H | 6.64 | C20H22O9 | 405.12 | -1.36 |  | ✔ | ✔ |
| 27 | Polygalic acid | M-H | 7.33 | C29H44O6 | 487.31 | -2.61 | ✔ | ✔ |  |
| 28 | 2''-O-beta-L-galactopyranosylorientin | M-H | 5.47 | C27H30O16 | 609.15 | 0.01 |  | ✔ |  |
| 29 | Gardenoside | M-H | 0.82 | C17H24O11 | 403.12 | -1.23 |  | ✔ | ✔ |
| 30 | Amygdalin | M-H | 8 | C20H27NO11 | 456.15 | -0.55 |  | ✔ |  |
| 31 | Arbutin | M-H | 1.05 | C12H16O7 | 271.08 | -0.31 |  | ✔ |  |
| 32 | Leucoside | M-H | 8.16 | C26H28O15 | 579.14 | 0.19 | ✔ |  |  |
| 33 | Astringin | M-H | 6.64 | C20H22O9 | 405.12 | -1.36 |  | ✔ | ✔ |
| 34 | Forsythoside E | M-H | 6.53 | C20H30O12 | 461.17 | -0.7 |  | ✔ |  |
| 35 | Picroside III | M-H | 1.27 | C25H30O13 | 537.16 | -0.42 |  | ✔ | ✔ |
| 36 | Cynaroside | M-H | 8.65 | C21H20O11 | 447.09 | 0.02 | ✔ |  |  |
| 37 | Uridine | M-H | 1.04 | C9H12N2O6 | 243.06 | -0.52 | ✔ |  | ✔ |
| 38 | (2R,3S)-3-Phenylisoserine hydrochloride | M-H | 1.05 | C9H11NO3HCl | 180.07 | -0.68 |  | ✔ | ✔ |
| 39 | L-Tyrosine | M-H | 1.05 | C9H11NO3 | 180.07 | -0.68 |  | ✔ | ✔ |
| 40 | L-Glutamic acid | M-H | 0.73 | C5H9NO4 | 146.05 | 0.13 | ✔ | ✔ |  |
| 41 | 4-Hydroxyisoleucine | M-H | 0.8 | C6H13NO3 | 146.08 | 1.53 | ✔ |  |  |
| 42 | L-Theanine | M-H | 0.8 | C7H14N2O3 | 173.09 | -1.41 | ✔ | ✔ |  |
| 43 | L-Hydroxyproline | M-H | 0.77 | C5H9NO3 | 130.05 | -1.1 | ✔ | ✔ | ✔ |
| 44 | Propylparaben | M-H | 1.29 | C10H12O3 | 179.07 | -0.41 | ✔ |  |  |
| 45 | Propyl gallate | M-H | 2.61 | C10H12O5 | 211.06 | -4.54 | ✔ |  |  |
| 46 | Nicotinic acid | M-H | 0.82 | C6H5NO2 | 122.02 | -2.1 | ✔ | ✔ |  |
| 47 | Polydatin | M-H | 7.79 | C20H22O8 | 389.12 | -1.21 |  | ✔ | ✔ |
| 48 | 1-Caffeoylquinic acid | M-H | 1.05 | C16H18O9 | 353.09 | -1.33 |  | ✔ | ✔ |
| 49 | Chrysophanol 8-O-beta-D-glucoside | M-H | 9.34 | C21H20O9 | 415.1 | -0.23 |  |  | ✔ |
| 50 | p-Hydroxybenzaldehyde | M-H | 5.46 | C7H6O2 | 121.03 | -0.04 | ✔ | ✔ |  |
| 51 | Chlorogenic acid | M-H | 1.05 | C16H18O9 | 353.09 | -1.33 |  | ✔ | ✔ |
| 52 | Cryptochlorogenic acid | M-H | 1.05 | C16H18O9 | 353.09 | -1.33 |  | ✔ | ✔ |
| 53 | Ferulic acid | M-H | 1.05 | C10H10O4 | 193.05 | -1.2 |  |  | ✔ |
| 54 | Shikimic acid | M-H | 0.82 | C7H10O5 | 173.05 | -0.72 |  | ✔ | ✔ |
| 55 | Isoferulic acid | M-H | 1.05 | C10H10O4 | 193.05 | -1.2 |  |  | ✔ |
| 56 | p-Coumaric acid | M-H | 1.33 | C9H8O3 | 163.04 | -0.66 | ✔ | ✔ | ✔ |
| 57 | p-Hydroxy-cinnamic acid | M-H | 1.33 | C9H8O3 | 163.04 | -0.66 | ✔ | ✔ | ✔ |
| 58 | Orsellinic acid | M-H | 1.05 | C8H8O4 | 167.03 | -0.68 | ✔ | ✔ |  |
| 59 | (R)-Mandelic acid | M-H | 9.08 | C8H8O3 | 151.04 | -0.81 | ✔ |  | ✔ |
| 60 | Quinic acid | M-H | 0.73 | C7H12O6 | 191.06 | -1.3 | ✔ | ✔ | ✔ |
| 61 | 4-Methoxysalicylic acid | M-H | 1.04 | C8H8O4 | 167.03 | -0.68 | ✔ | ✔ |  |
| 62 | Sucrose | M-H | 0.79 | C12H22O11 | 341.11 | -1.83 | ✔ |  | ✔ |
| 63 | 5-Acetylsalicylic acid | M-H | 1.05 | C9H8O4 | 179.03 | -0.89 |  | ✔ | ✔ |
| 64 | L-Tyrosine | M-H | 1.05 | C9H11NO3 | 180.07 | -0.68 |  | ✔ | ✔ |
| 65 | Gentiopicrin | M-H | 0.84 | C16H20O9 | 355.1 | 0.71 |  |  | ✔ |
| 66 | Orcinol gentiobioside | M-H | 5.99 | C19H28O12 | 447.15 | 0.39 |  |  | ✔ |
| 67 | Esculin | M-H | 2.89 | C15H16O9 | 339.07 | -0.05 |  |  | ✔ |
| 68 | Specnuezhenide | M-H | 17.92 | C31H42O17 | 685.23 | 0.48 |  |  | ✔ |
| 69 | Secoxyloganin | M-H | 4.87 | C17H24O11 | 403.12 | -1.23 |  |  | ✔ |
| 70 | Scutellarin | M-H | 8.01 | C21H18O12 | 461.07 | 0.71 |  |  | ✔ |
| 71 | Eriocitrin | M-H | 5.16 | C27H32O15 | 595.17 | 0.05 |  |  | ✔ |
| 72 | Chikusetsu saponin 4a | M-H | 19.82 | C42H66O14 | 793.44 | -2.68 |  |  | ✔ |
| 73 | Bilobalide | M-H | 1.04 | C15H18O8 | 325.09 | 0.86 |  |  | ✔ |
| 74 | Apigenin 7-glucuronide | M-H | 7.18 | C21H18O11 | 445.08 | 0.5 |  |  | ✔ |
| 75 | Baicalin | M-H | 7.18 | C21H18O11 | 445.08 | 0.5 |  |  | ✔ |
| 76 | Luteolin 7-glucuronide | M-H | 8.01 | C21H18O12 | 461.07 | 0.71 |  |  | ✔ |
| 77 | Taxifolin 7-rhamnoside | M-H | 8.11 | C21H22O11 | 449.11 | 1.81 |  |  | ✔ |
| 78 | Engeletin | M-H | 9 | C21H22O10 | 433.11 | 0.7 |  |  | ✔ |
| 79 | Quercetin 7-rhamnoside | M-H | 8.65 | C21H20O11 | 447.09 | 0.02 | ✔ |  |  |
| 80 | Ethyl ferulate | M-H | 6.75 | C12H14O4 | 221.08 | -0.04 | ✔ |  |  |
| 81 | Vanillin | M-H | 9.08 | C8H8O3 | 151.04 | -0.81 | ✔ | ✔ |  |
| 82 | Specnuezhenide | M-H | 17.92 | C31H42O17 | 685.23 | 0.48 |  |  | ✔ |
| 83 | Polydatin | M-H | 7.79 | C20H22O8 | 389.12 | -1.21 |  | ✔ | ✔ |
| 84 | Bilobalide | M-H | 1.04 | C15H18O8 | 325.09 | 0.86 |  |  | ✔ |
| 85 | Ginkgolide K | M-H | 6.64 | C20H22O9 | 405.12 | -1.36 |  | ✔ | ✔ |
| 86 | Quinic acid | M-H | 0.73 | C7H12O6 | 191.06 | -1.3 | ✔ | ✔ | ✔ |
| 87 | Citric acid | M-H | 0.72 | C6H8O7 | 191.02 | -0.32 |  | ✔ | ✔ |
| 88 | Maleic acid | M-H | 0.72 | C4H4O4 | 115 | 0.24 |  | ✔ | ✔ |
| 89 | Fumaric acid | M-H | 0.72 | C4H4O4 | 115 | 0.24 |  | ✔ | ✔ |
| 90 | p-Hydroxy-cinnamic acid | M-H | 1.33 | C9H8O3 | 163.04 | -0.66 | ✔ | ✔ | ✔ |
| 91 | p-Coumaric acid | M-H | 1.33 | C9H8O3 | 163.04 | -0.66 | ✔ | ✔ | ✔ |
| 92 | Ethyl gallate | M-H | 1.05 | C9H10O5 | 197.05 | -0.63 |  | ✔ |  |
| 93 | Chlorogenic acid | M-H | 1.05 | C16H18O9 | 353.09 | -1.33 |  | ✔ | ✔ |
| 94 | Mannitol | M-H,M+Cl- | 0.77 | C6H14O6 | 181.07 | -1.08 |  | ✔ |  |
| 95 | p-Hydroxybenzaldehyde | M-H | 5.46 | C7H6O2 | 121.03 | -0.04 | ✔ | ✔ |  |
